# Seed sequence polymorphism rs2168518 and allele-specific target gene regulation of hsa-miR-4513

**DOI:** 10.1093/hmg/ddab292

**Published:** 2021-10-04

**Authors:** Christina Kiel, Tobias Strunz, Daniele Hasler, Gunter Meister, Felix Grassmann, Bernhard H F Weber

**Affiliations:** Institute of Human Genetics, University of Regensburg, Regensburg 93053, Germany; Institute of Human Genetics, University of Regensburg, Regensburg 93053, Germany; Regensburg Center for Biochemistry (RCB), Laboratory for RNA Biology, University of Regensburg, Regensburg 93053, Germany; Regensburg Center for Biochemistry (RCB), Laboratory for RNA Biology, University of Regensburg, Regensburg 93053, Germany; Institute of Human Genetics, University of Regensburg, Regensburg 93053, Germany; Institute of Medical Sciences, University of Aberdeen, King’s College, Aberdeen AB24 3FX, UK; Institute of Human Genetics, University of Regensburg, Regensburg 93053, Germany; Institute of Clinical Human Genetics, University Hospital Regensburg, Regensburg 93053, Germany

## Abstract

MicroRNAs (miRNAs) are small post-transcriptional regulators that offer promising targets for treating complex diseases. To this end, hsa-miR-4513 is an excellent candidate as this gene harbors within its conserved heptametrical seed sequence a frequent polymorphism (rs2168518), which has previously been associated with several complex phenotypes. So far, little is known about the biological mechanism(s) underlying these associations. In an initial step, we now aimed to identify allele-specific target genes of hsa-miR-4513. We performed RNA sequencing in a miRNA overexpression model in human umbilical vein endothelial cells transfected with separated hsa-miR-4513 alleles at rs2168518, namely hsa-miR-4513-G and hsa-miR-4513-A. Genes specifically regulated by the rs2168518 alleles were independently verified by quantitative reverse transcription PCR (qRT-PCR), western blot analysis and allele-specific miRNA binding *via* a luciferase reporter assay. By a text-based search publicly available databases such as Online Mendelian Inheritance in Man and Mouse Genome Informatics were utilized to link target genes of hsa-miR-4513 to previously described phenotypes. Overall, we identified 23 allele-specific hsa-miR-4513 target genes and replicated 19 of those independently *via* qRT-PCR. Western blot analysis and luciferase reporter assays conducted for an exemplary subsample further confirmed the allele-specific regulation of these genes by hsa-miR-4513. Remarkably, multiple allele-specific target genes identified are linked via text retrieval to several phenotypes previously reported to be associated with hsa-miR-4513. These genes offer promising candidates for ongoing research on the functional pathobiological impact of hsa-miR-4513 and its seed polymorphism rs2168518. This could give rise to therapeutic applications targeting this miRNA.

## Introduction

Understanding the pathobiology of complex diseases is of utmost priority when aiming for therapeutic intervention targeting specific pathological processes. To this end, biomarkers have gained particular interest not only as disease predictors but also as targets for individualized therapies. Specifically, microRNAs (miRNAs) are attractive potential biomarkers as their expression pattern can reflect an underlying pathophysiology potentially specific to various disease states (1–4).

When fully matured, miRNAs are small non-coding RNAs of ~22 nucleotides in length. After being processed and integrated into the RNA-induced silencing complex, they reveal their effect on post-transcriptional gene expression regulation by guiding the complex to their specific target transcripts. Recognition and binding to the target mRNA is achieved *via* complementary base pairing, for which the seed region, spanning nucleotides 2–7 at the 5′-end of the miRNA, plays a crucial role (5,6). Genetic polymorphisms located in this sequence of miRNAs are rare but are expected to have strong effects on the post-transcriptional regulation mediated by these regulators and thus may vary in their allelic contribution to phenotype expression (7).

Hsa-miR-4513 is an exemplary miRNA harboring a common polymorphism (rs2168518) in its seed region and was recently reported to be overexpressed in several cancer cell lines (8–11) as well as in cancer tissue (11). In addition, rs2168518 was associated with several clinically relevant traits and diseases, including cardiovascular phenotypes (12–15), fasting glucose and lipid traits (12), age-related macular degeneration (15,16) and different metabolic products in urine (15). Moreover, rs2168518 is suggested as possible biomarker to predict lung adenocarcinoma survival rates after treatment with tyrosine kinase inhibitors (17). These findings highlight the potential clinical relevance of hsa-miR-4513 and demonstrate the need to gain deeper insight into its underlying functional role, specifically in relation to its seed polymorphism rs2168518.

Previous studies have suggested target genes of hsa-miR-4513 for selected phenotypes mainly by bioinformatical approaches, and most often without considering the influence of seed polymorphism rs2168518 (8–12,16). Here, we used a global RNA-sequencing (RNA-Seq) approach to determine target genes of hsa-miR-4513, specifically considering the influence of seed polymorphism rs2168518 on target binding. We validated the findings by independent replication, western blot analysis and luciferase reporter assays, the latter confirming allele-specific binding of hsa-miR-4513 to the target gene sequences. A text-based search collated data with phenotypes formerly reported in the context of hsa-miR-4513 suggesting functional roles of allele-specific target genes in disease processes.

## Results

### Identification of hsa-miR-4513 target genes

To determine target genes of hsa-miR-4513, we performed RNA-Seq in a cellular miRNA overexpression model using primary endothelial cells due to their relevance in cardiovascular conditions as well as in neovascular age-related macular degeneration followed by multiple validation approaches (Supplementary Material, Fig. S1). To this end, human umbilical vein endothelial cells (HUVECs) were transfected with hsa-miR-4513-G (ancestral allele at rs2168518), hsa-miR-4513-A (minor allele at rs2168518) or the control miRNA cel-miR-39, the latter having no homologous sequence in human. Transfection efficiency was validated *via* quantitative reverse transcription PCR (qRT-PCR). For the three miRNAs a robust increased miRNA expression was observed 48 h after transfection, ranging from a 676-fold to a 1458-fold upregulation compared with control (Supplementary Material, Fig. S2A). Differences in the relative expression after transfection can be explained by the fact that transfection efficiency was normalized to endogenous miRNA expression, whereas the control miRNA cel-miR-39 has no homologue in the human DNA. Considering the absolute cycle threshold (Ct) values of the qRT-PCRs the expression after transfection is comparable between all miRNAs (mean Ct-value with standard deviation of hsa-miR-4513-A = 16.72 ± 0.47, hsa-miR-4513-G = 16.35 ± 0.89 and cel-miR-39 = 15.19 ± 0.88).

**Figure 1:**
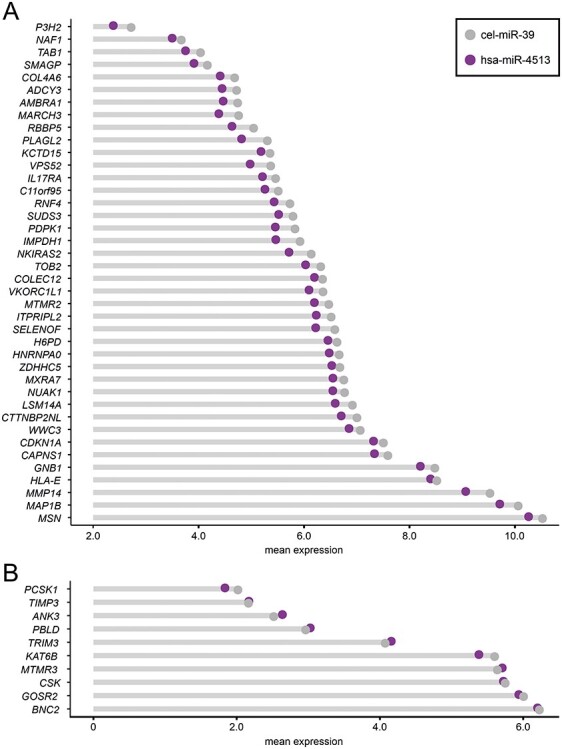
A lollipop plot representation of target genes of hsa-miR-4513. (**A**) Displayed are target genes of hsa-miR-4513 identified *via* RNA-sequencing (RNA-Seq) of human umbilical vein endothelial cells (HUVECs) transfected with hsa-miR-4513-A, hsa-miR-4513-G or cel-miR-39 as control. In total, 40 genes showed a significantly decreased expression between hsa-miR-4513 (both alleles combined, purple) and the control samples (grey) [false discovery (FDR) corrected *P*-value (*Q*-value) < 0.01]. (**B**) Expression of hsa-miR-4513 target genes previously identified in the literature (8–12,16), as well as the host gene *CSK* (12) in the RNA-Seq data of HUVECs transfected with hsa-miR-4513-A, hsa-miR-4513-G and cel-miR-39 as control. Significant expression differences between the control cel-miR-39 and hsa-miR-4513 samples were only observed for *KAT6B* (*Q*-value < 0.05). Expression values of target genes are shown separately for all groups investigated in Supplementary Material, Figure S3.

For a global identification of hsa-miR-4513 target genes in RNA-Seq data, data from hsa-miR-4513-G and hsa-miR-4513-A were initially combined. In total, we identified 40 protein-coding target genes of hsa-miR-4513 with a significantly decreased expression after hsa-miR-4513 transfection [false discovery rate (FDR) corrected, *Q*-value < 0.01; Fig. 1A, (Supplementary Material, Fig. S3A and Supplementary Material, Table S1)]. Enrichment analysis of these genes (18) revealed no significant biological pathway (adjusted *P*-value of the g:Profiler web tool < 0.05), emphasizing that potential target genes likely exhibit various functions.

Parallel to the undirected RNA-Seq approach, genes previously linked to hsa-miR-4513 were included in the study. To this end, we conducted a literature search in the context of specific phenotypes, including cardiometabolic phenotypes (12), age-related macular degeneration (16) and several types of cancer (8–11). Further, one study reported that the seed polymorphism rs2168518 in hsa-miR-4513 revealed an effect on expression of *CSK*, the host gene of hsa-miR-4513 (12). In total, 11 genes were identified. One of these, *CXCL17*, failed to reach our specified expression thresholds and was therefore excluded. Of the remaining 10 genes only 2 showed significant expression differences between the investigated groups, including *KAT6B* [FDR corrected, *Q*-value = 0.042 between combined hsa-miR-4513 and control; Fig. 1B, Supplementary Material, Fig. S3B and Supplementary Material, Table S2] and *MTMR3* [FDR corrected, *Q*-value = 0.029 between hsa-miR-4513-A and hsa-miR-4513-G; Supplementary Material, Fig. S3B and Supplementary Material, Table S2]. Although *MTMR3* expression displayed significant differences between the hsa-miR-4513 alleles, there was no significant difference in comparison with the control group.

### Allele-specific target genes of hsa-miR-4513

**Figure 2:**
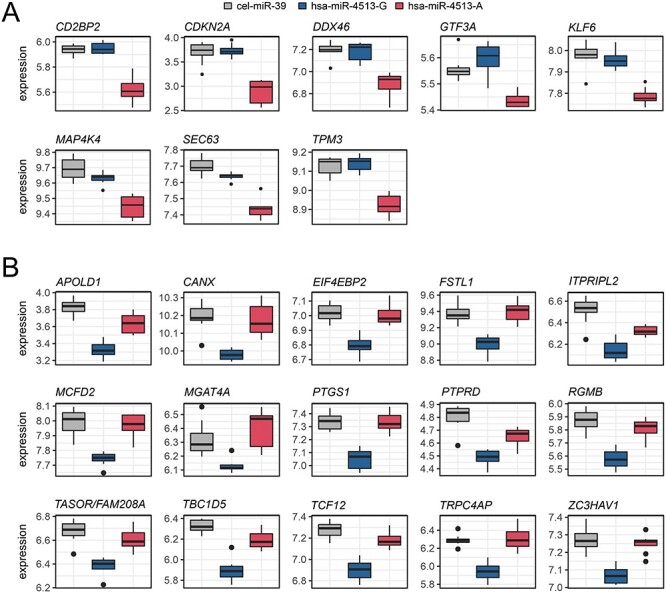
Allele-specific target genes of hsa-miR-4513. Boxplot representations of expression of allele-specific target genes of (**A**) hsa-miR-4513-G and (**B**) has-miR-4513-A identified in the RNA-Seq data of HUVECs transfected with hsa-miR-4513-A, hsa-miR-4513-G and cel-miR-39 as control. Allele-specific target genes revealed significantly decreased expression for hsa-miR-4513 with the respective allele in comparison with cel-miR-39 and a significantly altered expression between the two rs2168518 alleles (both *Q*-values < 0.01). In total, 15 genes were considered allele-specific target genes of hsa-miR-4513-G and 8 genes as allele-specific target genes of hsa-miR-4513-A. Expression values represent estimated gene expression counts, converted to log2 transformed CPM.

Several studies have reported an association of the hsa-miR-4513 seed polymorphism rs2168518 with diverse phenotypes (12–17). We screened the RNA-Seq data for genes, which displayed a significantly altered expression between hsa-miR-4513-A and hsa-miR-4513-G (FDR corrected, *Q*-value < 0.01). Furthermore, candidate genes for the respective allele had to show a significantly decreased expression in comparison with control (transcriptome wide correction by FDR, *Q*-value < 0.01) to be considered a direct target gene. This resulted in 8 potential target genes of the hsa-miR-4513-A allele and 15 potential target genes of the hsa-miR-4513-G allele (Fig. 2 and Supplementary Material, Table S3).

**Figure 3:**
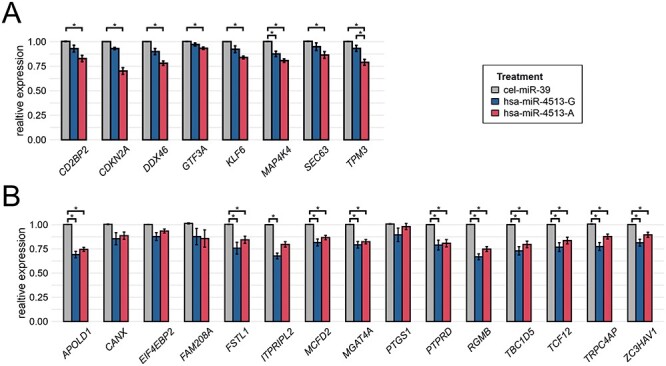
Validation of allele-specific hsa-miR-4513 target genes. Allele-specific target genes of hsa-miR-4513 were independently validated *via* qRT-PCR after transfection of HUVECs with hsa-miR-4513-A, hsa-miR-4513-G or the control cel-miR-39. The relative expression was normalized to cel-miR-39 transfected samples. (**A**) Allele-specific target genes of hsa-miR-4513-G and (**B**) target genes of hsa-miR-4513-A. Shown are mean values from five to six independent experiments, with two to three replicates each. Bars indicate standard error (SE). Statistical significance was determined with the Kruskal–Wallis test followed by a Dunn’s multiple comparison test to adjust for multiple testing. ^*^ Adjusted *P*-value < 0.05.

Genes that displayed a potential allele-specific regulation in the RNA-Seq data were independently validated *via* qRT-PCR in HUVECs. To this end, RNA was isolated from independently transfected cells. Overall, replication was achieved for all allele-specific candidates (Fig. 3) although not all reached statistical significance (Kruskal–Wallis test, *P*-value < 0.05). Eleven of the 15 potential target genes of hsa-miR-4513-G showed a significantly reduced expression in hsa-miR-4513-G transfected samples when compared with control samples [Dunn’s multiple comparison test, adjusted *P*-value < 0.05; Fig. 3A and Supplementary Material, Table S4]. Ten of the 11 hsa-miR-4513-G target genes showed also a significant difference in expression between hsa-miR-4513-A and the control group, but none displayed a significant difference between hsa-miR-4513-G and hsa-miR-4513-A. In contrast, all of the eight target genes of hsa-miR-4513-A were validated *via* qRT-PCR with significantly reduced expression in hsa-miR-4513-A samples in comparison with control (Fig. 3B). Only one of those genes, namely *MAPAK4*, was significantly less expressed in hsa-miR-4513-G in comparison with the control samples (adjusted *P*-value = 0.030). Further, *TPM3* was the only gene with a significantly altered expression between hsa-miR-4513-A and hsa-miR-4513-G (adjusted *P*-value = 0.021).

### Protein expression of allele-specific hsa-miR-4513 target genes

Six allele-specific hsa-miR-4513 target genes were selected for analysis *via* western blot and luciferase reporter assay. Selection criteria included: (i) significant expression difference between the three investigated groups according to the Kruskal–Wallis test (*P*-value < 0.05), (ii) high expression in HUVECs (Ct-value <25), (iii) absolute expression difference between the hsa-miR-4513 samples with the respective allele and the control of >15% and (iv) significant expression difference for only one of the two hsa-miR-4513 alleles in comparison with the control group (Dunn’s multiple comparison test, adjusted *P*-value < 0.05). For western blot analysis, it was not feasible to obtain a specific protein staining for ITPRIPL2, the only target gene in our series specifically regulated by hsa-miR-4513-G. The five remaining target genes that met the aforementioned criteria are all specifically regulated by hsa-miR-4513-A, including CD2BP2, CDKN2A, DDX46, KLF6 and TPM3.

**Figure 4:**
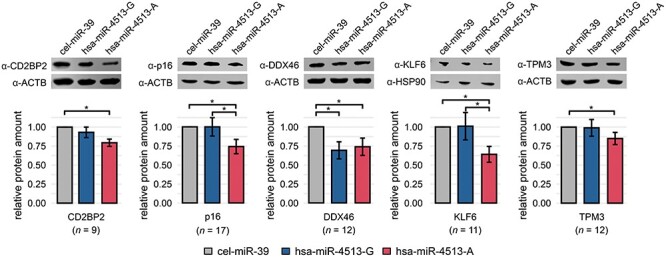
Allele-specific hsa-miR-4513 target gene protein expression. Representative western blot analyses of five selected target genes with the respective loading controls ACTB or HSP90 (for KLF6). Isoform p16 represents one of the protein products of *CDKN2A*. All samples were normalized to their respective loading control ACTB or HSP90 and the control miRNA cel-miR-39. Shown are mean values from 9 to 17 independent experiments. Bars indicate SE. Statistical significance was determined with the Kruskal–Wallis test followed by a Dunn’s multiple comparison test to adjust for multiple testing. ^*^ Adjusted *P*-value < 0.05.

An effect of hsa-miR-4513 and its seed polymorphism on the respective protein expression was investigated *via* western blot analysis. *CD2BP2*, *CDKN2A* (protein product p16), *DDX46*, *KLF6* and *TPM3* showed reduced protein expression in hsa-miR-4513-A transfected samples in comparison with the control group (Dunn’s multiple comparison test, adjusted *P*-value < 0.05; Fig. 4). Two proteins, p16 and KLF6, further displayed significant differences between the rs2168518 alleles in hsa-miR-4513 transfected samples (adjusted *P*-value = 0.042 and 0.044). DDX46 revealed a significantly reduced protein expression in hsa-miR-4513-A and hsa-miR-4513-G transfected samples in comparison with control (adjusted *P*-value = 0.032 and 0.023). While a detection of the protein expressed by *ITPRIPL2* was not feasible, most of the investigated genes and their respective protein products represent valid allele-specific target genes of hsa-miR-4513. An exception is DDX46, which is regulated by hsa-miR-4513 but shows no allele-specificity.

### Verification of allele-specific target genes of hsa-miR-4513 *via* luciferase reporter assay

**Figure 5:**
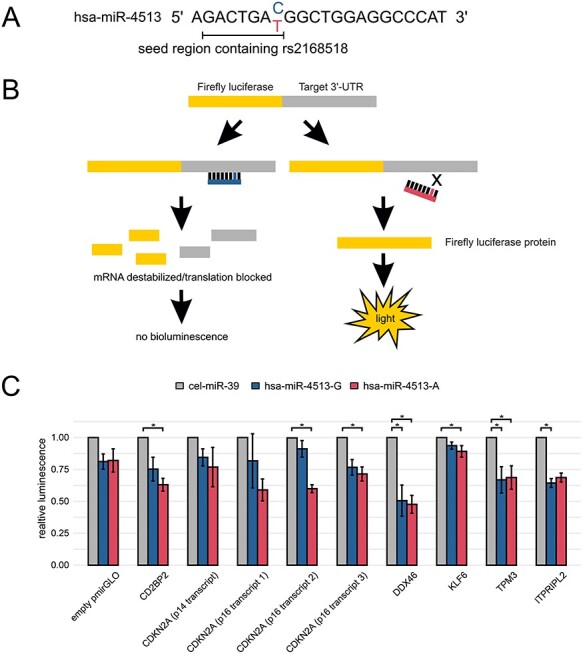
Verification of allele-specificity of hsa-miR-4513 target genes *via* luciferase reporter assay. (**A**) Mature miRNA sequence of hsa-miR-4513 with seed polymorphism rs2168518. (**B**) Schematic overview of the luciferase reporter assay. The 3′-untranslated regions (3′-UTR) of identified hsa-miR-4513 target genes were inserted into the pmirGLO reporter vector as the 3′-UTR of the firefly *luciferase* gene. If a specific binding site for the miRNA is present in the sequence, the miRNA binds to the luciferase mRNA transcript and leads to its destabilization or blocks its translation ultimately resulting in a reduced light signal. In the absence of a binding site, the firefly *luciferase* transcript can be translated into a protein and a bioluminescence signal is measured. (**C**) The 3′-UTRs of selected allele-specific target genes were introduced into the pmirGLO luciferase reporter vector and co-transfected with hsa-miR-4513-G, hsa-miR-4513-A or cel-miR-39 as control in HEK293T (human embryonic kidney) cells. Twenty-four hours after transfection, the luciferase luminescence signal was measured and normalized to the cel-miR-39 transfected cells. For the *CDKN2A* gene four transcripts, all detectable in the primary RNA-Seq analysis in HUVECs, were analyzed. Shown are mean values from four independent experiments, with four to six replicates each. Bars indicate SE. Statistical significance was determined with the Kruskal–Wallis test followed by a Dunn’s multiple comparison test to adjust for multiple testing. ^*^ Adjusted *P*-value < 0.05.

Specific binding by the rs2168518A or rs2168518G allele of hsa-miR-4513 (Fig. 5A), was investigated by a luciferase reporter assay. The reporter vector contains a firefly *luciferase* coding region combined with the 3′-untranslated region (3′-UTR) of the respective target gene transcript (Fig. 5B). It should be mentioned that binding to the 3′-UTR of target genes is the most common but not the only way that miRNAs can regulate their target genes (6). Upon specific miRNA binding to the 3′-UTR, the luminescence signal emitted by the luciferase is diminished. In contrast, if there is no suitable miRNA binding site within the 3′-UTR, the luminescence signal remains unaltered. UCSC Genome Browser database entries show that the 3′-UTRs of the genes investigated all contain allele-specific binding sites for hsa-miR-4513 (Supplementary Material, Fig. S4). Moreover, the 3′-UTRs of four of the six genes contain binding sites, which perfectly match the seed region of hsa-miR-4513. The remaining two genes display binding sites in their 3′-UTR, which extend the seed region of the miRNA but still include the position of the polymorphic site. These allele-specific binding sites are in full agreement with our results of allele-specific mRNA repression. Nevertheless, in most 3′-UTRs less specific binding sites for hsa-miR-4513 are present by prediction, which either may not show allele-specificity for seed polymorphism rs2168518 or comprise <7 nucleotides for binding.

To ensure reliability of the assay, a reporter vector with an internal transfection control was used. The firefly luminescence signal was normalized for transfection efficiency by a second luminescence signal, the *Renilla* luminescence signal, which is independent from miRNA binding. Further, successful co-transfection of the miRNA mimics was ensured by qRT-PCR analysis (Supplementary Material, Fig. S2). We also tested the binding behavior of the tested miRNAs (hsa-miR-4513-A, hsa-miR-4513-G or cel-miR-39) on the empty reporter vector pmirGLO. Cells co-transfected with hsa-miR-4513 showed a slightly lower luminescence compared with cells transfected with the miRNA control, independent of the rs2168518 polymorphism, although this observation was not statistically significant [Dunn’s multiple comparison test, adjusted *P*-value > 0.05; Fig. 5C]. In contrast, for all genes investigated a significant attenuation of luminescence occurred when transfected with one of the hsa-miR-4513 alleles in comparison with control miRNA (Dunn’s multiple comparison test, adjusted *P*-value < 0.05). Samples transfected with 3′-UTR sequences of *DDX46* and *TPM3* were significantly reduced when co-transfected with both hsa-miR-4513 variants in comparison with the control (*DDX46* hsa-miR-4513-A versus control adjusted *P*-value = 0.038 and hsa-miR-4513-G versus control adjusted *P*-value = 0.033; *TPM3* hsa-miR-4513-A versus control adjusted *P*-value = 0.038 and hsa-miR-4513-G versus control adjusted *P*-value = 0.033). For *CDKN2A*, two of four 3′-UTRs investigated showed a significant reduction of luminescence when transfected with hsa-miR-4513-A in comparison with control (p16 transcript 2 adjusted *P*-value = 0.016 and p16 transcript 3 adjusted *P*-value = 0.021), whereas the two remaining 3′-UTRs failed to show significance. Overall, the luciferase reporter assay corroborates the results of the earlier analyses and reinforces that the seed polymorphism of hsa-miR-4513 influences gene expression in an allele-specific manner.

### Clinical relevance of allele-specific target genes of hsa-miR-4513

**Figure 6:**
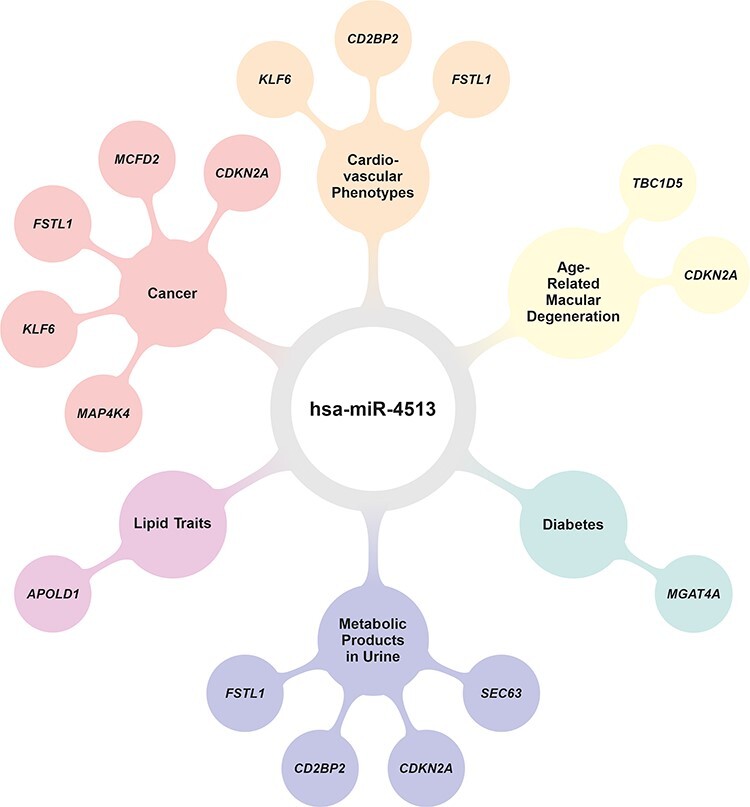
Results of literature text searches relating allele-specific hsa-miR-4513 target genes with phenotypes. Various phenotypes have been reported in the literature to which hsa-miR-4513 or its seed polymorphism rs2168518 have been linked, including cancer (8–11,17), cardiovascular phenotypes (12–15), age-related macular degeneration (15,16), diabetes (12), metabolic products in urine (15) and several lipid traits (12). The online databases OMIM (19) and MGI (22) were searched to assign allele-specific target genes to individual phenotype groups by specific keywords defined as follows: ‘cell proliferation’ or ‘cell death’ (cancer), ‘cardiovascular system’ (cardiovascular phenotypes), ‘vision/eye’ (age-related macular degeneration), ‘cellular glucose’ or ‘insulin resistance’ (diabetes), ‘homeostasis/metabolism’ and ‘renal/urinary system’ (metabolic products in urine) and ‘lipid binding’ (lipid traits). The graphic was created with LaTeX.

To learn about a clinical context of the potentially allele-specific target genes of hsa-miR-4513, we searched in publicly available databases such as Online Mendelian Inheritance in Man (OMIM) database (19), the genome aggregation database [gnomAD; (20,21)] and Mouse Genome Informatics (MGI) database (22) for phenotype-related key words (Supplementary Material, Table S5). Even though there is no obvious homolog for hsa-miR-4513 in mice, we have searched the MGI database to undergo a comprehensive assessment of the medical relevance of genes regulated by this or a related miRNA. We included all 19 potentially allele-specific target genes of hsa-miR-4513 in this analysis defined by a significantly reduced expression after hsa-miR-4513 transfection in the qRT-PCR validation analysis (adjusted *P*-value < 0.05). Of these, 10 were assigned to clinically relevant traits and phenotypes, whereby multiple assignments were possible (Fig. 6).

## Discussion

Over the past few years, a number of studies supported a clinical relevance of human miRNA hsa-miR-4513 and its seed polymorphism rs2168518 (8–17). Against this background, we aimed to identify allele-specific target genes of hsa-miR-4513 by applying an undirected RNA-Seq approach. Primary endothelial cells transfected with the allelic seed sequence variants of hsa-miR-4513 were considered an adequate model. Overall, we identified 15 specific target genes of the hsa-miR-4513-G allele and eight specific target genes of the hsa-miR-4513-A allele. An independent validation of the findings confirmed 11 targets of hsa-miR-4513-G and the eight target genes of hsa-miR-4513-A. In addition, dual-luciferase reporter assays of the 3′-UTRs of selected allele-specific hsa-miR-4513 target genes further confirmed the strict binding specificity. Taken together, our results demonstrate that a number of human genes are regulated by hsa-miR-4513 in a strictly allele-specific fashion due to genetic variation at rs2168518 within the critical seed region of hsa-miR-4513.

A critical role for the interpretation of RNA-Seq results is attributed to data procession and especially normalization issues (23,24). For example, to overcome batch dependent variability in our RNA-Seq data, we performed several well-established normalization methods, including trimmed mean of *M*-values for read count normalization, quantile normalization to remove inter-sample variation, as well as ComBat to account for known batch effects (23–26). To verify the validity of our results, we relied on independent experimental validation of the allele-specific target genes and demonstrate a striking replication of effect direction and specificity of allele-specific binding to the 3′-UTRs of selected target genes. We also substantiated our findings by demonstrating gene expression regulation at the protein level.

Due to its likely clinical relevance, hsa-miR-4513 and its seed polymorphism rs2168518 have gained increasing interest and several studies have reported potential target genes of hsa-miR-4513 (8–12,16). Interestingly, nine target genes were described previously and were also found in our data set, but only two of these displayed significant differences in our study although only target gene *KAT6B* (9) was finally validated.

In contrast to previous studies, our approach to identify target genes of hsa-miR-4513 was undirected and aimed at allele-specific responses due to the polymorphic site rs2168518 within the seed region. Remarkably, multiple allele-specific target genes could be assigned well correlating with phenotypes reported earlier. This, however, can only be an initial assignment at the present time. For example, *DDX46* and *PTPRD* have been associated with several types of cancer in various studies (27–33), but in our study only a measure of intolerance against loss of function mutations provided by gnomAD indicated an involvement in core biological processes (20,21).

Although hsa-miR-4513 acts in an allele-specific manner on multiple genes, those effects were not necessarily observed with statistical significance in all experiments and assays conducted. In fact, a consistent and statistically significant allele-specificity was observed for three target genes, namely *CDKN2A*, *CD2BP2* and *KLF6*. Difficulties in the replication of allele-specificity may be attributable to differences in the cell lines used, namely HUVECs for target gene identification and validation and HEK293T cells for investigation of allele-specific miRNA binding, alternatively to the timing between transfection and cell harvest for the various tests. Of note, the high exogenous expression of hsa-miR-4513 is likely not comparable with physiological expression of this miRNA, which is usually expressed at much lower levels (8–11). In case both alleles allow binding to the target transcript, although with a varying degree of binding specificity, the high expression in our experiments might obscure allele-specific effects. To investigate the impact of the hsa-miR-4513 seed polymorphism in a physiological context, a dataset with a large sample size will be needed in which both endogenous miRNA and mRNA expression data are provided simultaneously. Such data are currently not available. Unfortunately, also not in a large dataset like the one provided by the genotype-tissue expression project as mature miRNAs are not covered in the respective expression sequence data.

An interesting allele-specific target gene of hsa-miR-4513 identified in this study is *CDKN2A*. Proteins encoded by *CDKN2A* include p14 and p16 and exhibit crucial functions in cell cycle regulation as well as tumor suppression (34,35). Consistent with its role in cancer, *Cdkn2a* knockout mice reveal a systemic phenotype affecting a variety of tissues and cellular processes (22). A complete loss of a gene, as described in the MGI database, as well as pathogenic mutations, which in the case of *CDKN2A* can lead to hereditary cancer in human (36), are expected to display much stronger effects than regulatory influences such as those exerted by hsa-miR-4513. Overall, the systemic effect of *CDKN2A* expression can be reconciled with the highly pleiotropic associations found for hsa-miR-4513 and its seed polymorphism rs2168518 (8–16). In our study, *CDKN2A* was downregulated in an allele-specific way by hsa-miR-4513-A. Interestingly, Ghanbari and colleagues reported a lower expression of hsa-miR-4513-A in comparison with hsa-miR-4513-G in a transient transfection model using HEK293 cells (12). Based on such findings, we suggest that the allele-specific downregulation of *CDKN2A* is diminished due to a lower expression of the hsa-miR-4513-A allele. Furthermore, studies have shown that p14 and p16 expression increases with increasing age (37) and that the presence of p16 in senescent cells is associated with age-related pathologies (38). As a consequence, small allele-specific regulatory effects may only become apparent with an increase in target gene expression or by effects accumulating with age. This would also fit with the associations found for hsa-miR-4513 and its seed polymorphism rs2168518 with age-related phenotypes such as cancer (8–11), cardiovascular conditions (12–15) and age-related macular degeneration (15,16). In summary, allele-specific regulation of *CDKN2A* by hsa-miR-4513-A is a promising mechanism that could explain the association of the miRNA locus with seemingly unrelated age-related pathologies.

It should be noted that the approach chosen in this study aimed to identify allele-specific target genes of hsa-miR-4513. According to our experimental design, however, no conclusions can be drawn about potential local effects of rs2168518, or other genetic variants in high linkage disequilibrium with rs2168518. The latter effects still could influence genes in proximity and may be causative for the association signals reported in previous studies (12–17). Another explanation for the associations detected at this locus could be an expression quantitative trait locus, where a polymorphism has a regulating effect on the expression of nearby genes, eventually by altering a transcription factor binding site. To this end, several studies have reported regulatory effects of rs2168518, or of highly linked variants, on several genes located within the same locus as hsa-miR-4513, including the miRNA host gene *CSK* (12,15,39–41). Due to the exogenously increased miRNA expression following our study design, it is difficult to speculate about a possible co-expression of hsa-miR-4513 and its host gene *CSK* and its consequences. As of now, it remains unclear which mechanism underlies the single phenotype associations and to which extend several mechanisms may be interconnected.

Taken together, we identified target genes of hsa-miR-4513 isoforms, which are attributable to allele-specific binding of seed polymorphism rs2168518 at the targeted sites. Several of these genes are known to be of biological and clinical relevance for complex phenotypes which have been associated with this miRNA in previous studies. We suggest that these genes are excellent candidates for further investigations into the biological mechanisms of hsa-miR-4513 and the impact of its seed polymorphism on different phenotypes.

## Materials and Methods

### Cell culture and miRNA mimic transfection

HUVECs (Life Technologies, Carlsbad, CA, USA) were cultivated in EBM Plus Basal Medium (Lonza, Basel, Switzerland) supplemented with EGM Plus SingleQuots (Lonza) without antibiotics. Cells (< passage 5) were transfected with miRCURY LNA miRNA mimics (Qiagen, Hilden, Germany) and HiPerFect (Qiagen). Oligonucleotide primer sequences for miRNA mimics were: hsa-miR-4513-A (5′-AGA CUG AUG GCU GGA GGC CCA U-3′), hsa-miR-4513-G (5′-AGA CUG ACG GCU GGA GGC CCA U-3′) and cel-miR-39-3p (5’-UCA CCG GGU GUA AAU CAG CUU G-3′). HEK293T (human embryonic kidney) cells (ATCC, Manassas, Virginia, USA) were cultivated in DMEM high glucose medium (Thermo Fisher Scientific, Waltham, MA, USA) containing 10% fetal calf serum (Thermo Fisher Scientific) and 100 U/ml penicillin/streptomycin (Thermo Fisher Scientific). Using lipofectamine 2000 (Thermo Fisher Scientific) HEK293T cells were co-transfected with miRNA mimics and the pmirGLO Dual-Luciferase miRNA Target Expression Vector (Promega Corporation, Madison, Wisconsin, USA).

### miRNA isolation and qRT-PCR to verify transfection efficiency

HUVECs were transfected in triplicates on 12-well plates, HEK293T cells on 96-well plates, eight wells per condition. MiRNA transfection efficiency in HEK293T cells was determined by co-transfection of empty pmirGLO vector. MiRNAs were isolated 24 h (for HEK293T combining four wells) or 48 h (HUVECs) after transfection using the mirVANA microRNA Isolation kit (Ambion, Waltham, MA, USA) according to the procedures for organic extraction and total RNA isolation. The reverse transcription of miRNAs was performed according to ref. (42) by using 500 ng (HUVECs) or 1 μg (HEK293T) of purified miRNAs. PolyA tailing was achieved by *E. coli* Poly(A) Polymerase I (Ambion) using Universal RT oligonucleotide primer 5′-AAC GAG ACG ACG ACA GAC TTT TTT TTT TTT TTT V-3′. Reverse transcription was performed with superscript III reverse transcriptase (Invitrogen). Up to 2.5 ng of cDNA were used per qRT-PCR reaction with Takyon™ Low ROX SYBR Master Mix (Eurogentec, Cologne, Germany). qRT-PCR Primers were designed for the mature miRNA sequences with a polyA overhang (43) (hsa-miR-4513-G: 5′-TGA CGG CTG GAG GCC CAT AAA A-3′; hsa-miR-4513-A: 5′-TGA TGG CTG GAG GCC CAT AAA A-3′; cel-miR-39: 5’-TCA CCG GGT GTA AAT CAG CTT GAA AA-3′) and for the Universal Primer [5′-AAC GAG ACG ACG ACA GAC TTT-3′]. qRT-PCR reactions were run on a QuantStudio 5 (Applied Biosystems, Waltham, MA, USA) in technical triplicates. Overexpression of hsa-miR-4513-A/-G was normalized to cel-miR-39 transfected samples as control, whereas overexpression of cel-miR-39 was normalized to hsa-miR-4513-A/-G transfected samples.

### Library preparation and RNA-sequencing

The whole transcriptome approach included 8 samples of transfected HUVECs per treatment (hsa-miR-4513-A, hsa-miR-4513-G and cel-miR-39), each in three independent experiments. RNA-Seq libraries were constructed with the NEXTflex Rapid Directional mRNA-Seq Kit (PerkinElmer, Waltham, MA, USA). Up to 100 ng of total RNA were enriched by poly(A) beads and library quality was verified by Agilent BioAnalyzer DNA High-Sensitivity Chips (Agilent Technologies, Santa Clara, CA, USA). RNA-Seq was performed at the Genomics Core Facility ‘KFB—Center of Excellence for Fluorescent Bioanalytics’ (Dr Thomas Stempfl, www.kfb-regensburg.de) generating on average 15 million reads per sample. The libraries were quantified using the KAPA Library Quantification Kit—Illumina/ABI Prism (Roche Sequencing Solutions, Pleasanton, CA, USA). Equimolar amounts of each library were sequenced on an Illumina NextSeq 500 instrument (Illumina, San Diego, CA, USA) controlled by the NextSeq Control Software v2.2.0 using one 75 Cycles High Output Kit with the single index, single-read run parameters. Image analysis and base calling resulted in .bcl files, which were converted into .fastq files with the bcl2fastq v2.18 software.

### RNA sequencing data analysis

RNA-Seq data processing was done as described previously (25). Briefly, the quality of RNA-Seq data was ensured by applying *FastQC* [version 0.11.5; (44)] and *MultiQC* [version 1.7.dev0; (45)] during all steps of analysis. Trimming of adaptor sequences was not required. Raw reads were aligned to a reference genome based on Ensembl version 97 [GRCh38.p13, including GENCODE version 31; (46)] using the star aligner [version 2.7.1a; (47)] with ENCODE standard options. The *RSEM* toolbox [version 1.3.1; (48)] was applied with standard options to receive estimated gene expression counts. Due to single-end reads additional options including the mean fragment length and the standard deviation of the fragment length were added to the default settings. We adopted these parameters as defined elsewhere from two different data sets, with a mean fragment length of 155.9 and a standard deviation of 56.2 (25). Estimated gene counts were normalized by applying a trimmed mean of M-Values algorithm using the *tmmnorm* function implemented in the *edgeR* package [version 3.16.5; (49)] in R (50). Expression values were converted to counts per million (CPM) and genes with CPM > 1 in 10% of the samples were further analyzed.

Principal component analysis was performed with the *prcomp* function implemented in R (Supplementary Material, Fig. S5). CPM values were log2 transformed with an offset of 1. A quantile normalization and ComBat (26) were done to remove batch effects originating from different days of cell transfection and RNA isolation. To correlate gene expression and miRNA mimic transfection, a linear regression model was applied by the *lm* function implemented in R with adjustment for transfection batches. Only protein coding genes were included. Results were corrected for multiple testing according to the Benjamini and Hochberg method [FDR; (51)] implemented in the *multtest* package (52). FDR values (*Q*-values) below 0.01 were considered significant.

Allele-specific target genes were defined as showing significant differential gene expression between hsa-miR-4513-A and hsa-miR-4513-G and a significantly reduced gene expression in comparison with cel-miR-39 samples. Enrichment analysis of target genes was performed with the g:Profiler web tool [version e100_eg47_p14_7733820, accession date: 08.10.2020; (53)] with preset settings.

### mRNA isolation and qRT-PCR to validate target genes

HUVECs were transfected in triplicates on 12-well plates. Total RNA was isolated with the PureLink™ RNA Mini-Kit (Thermo Fisher Scientific) according to the manufacturer’s protocol including an on-column DNase digestion (Qiagen). Up to 3 μg of total RNA were used for first-strand cDNA synthesis using the RevertAid™ Reverse Transcriptase and Random Hexamer Primers (Thermo Fisher Scientific). qRT-PCR primers were designed on the basis of the Roche Library Probes [Roche, Basel, Switzerland; Supplementary Material, Table S6]. Up to 50 ng of cDNA were used per qRT-PCR reaction with Takyon™ Low ROX Probe 2X MasterMix dTTP Blue (Eurogentec). qRT-PCR reactions were run on a QuantStudio 5 (Applied Biosystems) in technical duplicates or triplicates and data were analyzed according to the ΔΔCt method (54). *HPRT1* values were used for normalization. At least two replicates per treatment day were required to be included as an independent replicate. In the validation analysis of target genes five to six independent replicates were used.

### Western blot

HUVECs were transfected in duplicates on 6-well plates and replicates were combined to obtain total protein extracts by homogenization in 1 × PBS. Protein extracts were sheared on ice for 15 s at 37% amplitude using a Vibra-Cell sonicator (Sonics, Newtown, CT, USA) and mixed with Laemmli buffer (55). Proteins were separated by sodium dodecyl sulfate polyacrylamide gel electrophoresis on 10% (for DDX46), 12.5% (for CD2BP2, TPM3 and KLF6) and 15% (for p16) gels and transferred to Immobilon-P polyvinylidene difluoride membranes (Merck Millipore, Burlington, MA, USA).

Antibodies used and dilutions applied were as follows: CD2BP2 (PA5-59603, diluted 1:1250, Thermo Fisher Scientific), p16 INK4A (D7C1M, diluted 1:1000, Cell Signaling Technologies, Danvers, MA, USA), ITPRIPL2 (HPA042011, no specific protein detectable, Sigma-Aldrich, St. Louis, MO, USA), TPM3 (720 306, diluted 1:1000, Life Technologies), KLF6 (PA5–79560, diluted 1:1000, Life Technologies), DDX46 (PA5–57713, diluted 1:1000, Life Technologies), HSP90 (sc-13 119, diluted 1:5000 Santa Cruz, Dallas, TX, USA) and β-Actin (A5441, diluted 1:10 000, Sigma-Aldrich). Secondary anti-mouse and anti-rabbit lgG antibodies were obtained from Merck Chemicals GmbH (Schwalbach, Germany) and diluted 1:10 000. Incubation of primary antibodies was carried out at 4°C overnight and incubation of secondary antibodies was done for 4 h at room temperature. For visualization of signals, the Clarity™ Western ECL Substrate (Bio-Rad Laboratories, Hercules, CA, USA) and an Odyssey FC imager (LI-COR, Lincoln, NE, USA) were used. Signal intensities were quantified with the Image Studio software (Version 4.0, LI-COR Biosciences). Beta-Actin signals measured from the same blot were used for normalization with one exception. As KLF6 and β-Actin have almost identical molecular weights, HSP90 was used for the normalization of KLF6 to ensure the possibility of a normalization to signals on the same blot. For quantification, western blot analyses were performed in 9–17 independent replicates.

### Generation of luciferase reporter vectors

Sequences of the 3′-UTR of selected target genes were obtained from the UCSC Genome Browser [GRCh38; (56)], available at https://genome.ucsc.edu/. Sequences were downloaded with the UCSC Table Browser tool (57). The 3′-UTR sequences were amplified from cDNA obtained from untreated HUVECs or HEK293T cells by PCR with primers as given in Supplementary Material, Table S7. All 3′-UTR sequences obtained were inserted into the pmirGLO Dual-Luciferase miRNA Target Expression Vector (Promega Corporation).

### Dual-luciferase reporter assay

HEK293T cells were co-transfected with the reporter vectors and miRNA mimics in 96-well plates. Twenty-four hours after transfection the luciferase reporter assay was performed with the Dual-Glo® Luciferase Assay System (Promega Corporation) according to the manufacturer’s protocol. Untreated cells served as control for background signal. The firefly luminescence signal was corrected for transfection efficiency with the internal control, the *Renilla* luciferase activity. Six technical replicates were included for each condition. If required, up to 2 outliers of the 6 technical replicates were excluded. Four independent experiments were performed for each luciferase reporter vector.

### Statistical evaluation

For statistical evaluations a Kruskal–Wallis test was performed in R. To correct for multiple testing, a Dunn’s multiple comparison test applying the Benjamini Hochberg method implemented in the fisheries stock analysis (FSA) package v0.8.26 (58) in R was used. Cel-miR-39 transfected cells served as controls.

### Text-based searches for target genes of hsa-miR-4513

Information about allele-specific target genes of hsa-miR-4513 was obtained from publicly available databases, namely OMIM (19), gnomAD (20,21) and MGI (22). An assignment of genes to clinical phenotypes was achieved with defined key words: (i) Cancer subtypes in OMIM or key words ‘cell proliferation’ or ‘cell death’ (MGI); (ii) Cardiovascular phenotypes in MGI mouse phenotypes by ‘cardiovascular system’; (iii) Age-related macular degeneration in MGI mouse phenotypes by ‘vision/eye’; (iv) Diabetic phenotypes by ‘cellular glucose’ or ‘insulin resistance’ (MGI); (v) Metabolic products in urine in MGI mouse phenotypes by ‘homeostasis/metabolism’ and ‘renal/urinary system’ and (vi) Lipid traits in Gene Ontology function implemented in MGI by ‘lipid binding’.

## Authors’ contributions

C.K. participated in study design, data acquisition, analysis and interpretation, visualized data and wrote the initial draft of the manuscript. T.S. performed RNA-sequencing data procession, contributed to data analysis and interpretation and critically revised the manuscript. D.H. substantially contributed to study conception and design, was involved in testing experimental approaches and critically revised the manuscript. G.M. substantially contributed to study conception and design, and critically revised the manuscript. F.G. acquired funding, participated in conceptualization and study design and critically revised the manuscript. B.H.F.W. participated in conceptualization and study design, participated in the interpretation of results, supervised and administrated the project and critically revised the manuscript. All authors have read and agreed to the published version of the manuscript.

## Supplementary Material

Supplemental_Figures_final_ddab292

Supplementary_Tables_final_ddab292
