# Supplementary material for: Seed sequence polymorphism rs2168518 and allele-specific target gene regulation of hsa-miR-4513
Source: Hum Mol Genet. 2021 Oct 4;31(6):875–87. doi: 10.1093/hmg/ddab292 (PMC8947236; doi:10.1093/hmg/ddab292)
Supplement: Supplemental_Figures_final_ddab292 [file supplemental_figures_final_ddab292.docx]

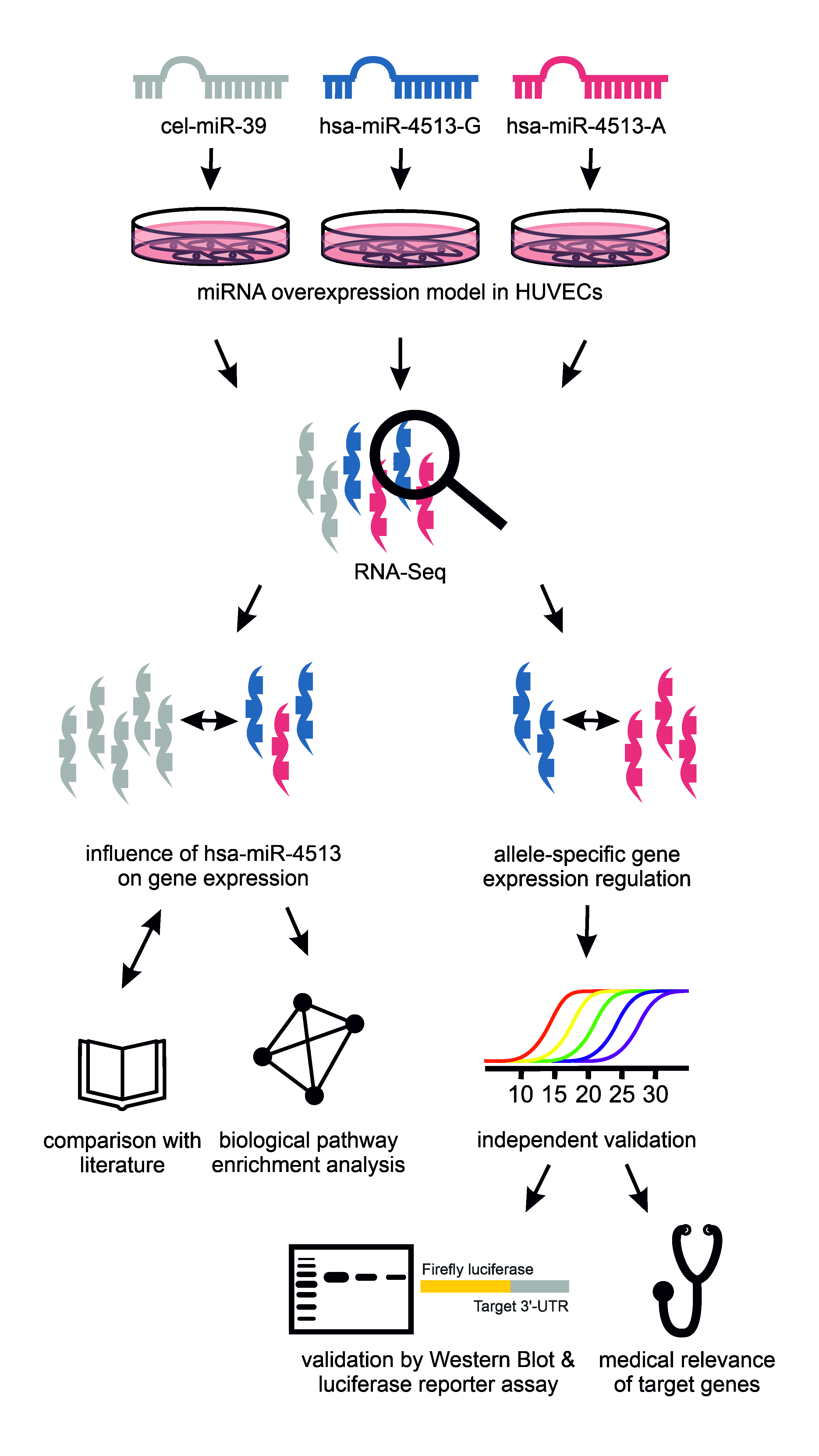


**Figure S1. Cartoon - study design.** Human umbilical vein endothelial cells (HUVECs) were transfected with control microRNA (miRNA) cel-miR-39 or mimics for hsa-miR-4513 harboring the A or G allele of the seed polymorphism rs2168518. Total RNA was isolated and sequenced (RNA-Seq). Genes with differential expression between hsa-miR-4513 transfected samples (both rs2168518 alleles combined) and cel-miR-39 transfected samples were classified as direct target genes of hsa-miR-4513 but without considering effects of seed polymorphism rs2168518. Additional target genes of hsa-miR-4513 were selected from the literature. Candidates were subjected to biological pathway enrichment analysis. In another line of investigations, allele-specific target genes of hsa-miR-4513 were determined from the RNA-Seq data. Candidate genes were independently validated by quantitative reverse transcription PCR (qRT-PCR). Furthermore, selected candidate genes were validated by Western Blot analysis and luciferase reporter assay. Allele-specific target genes were screened for their clinical relevance by text searches in publicly available databases.


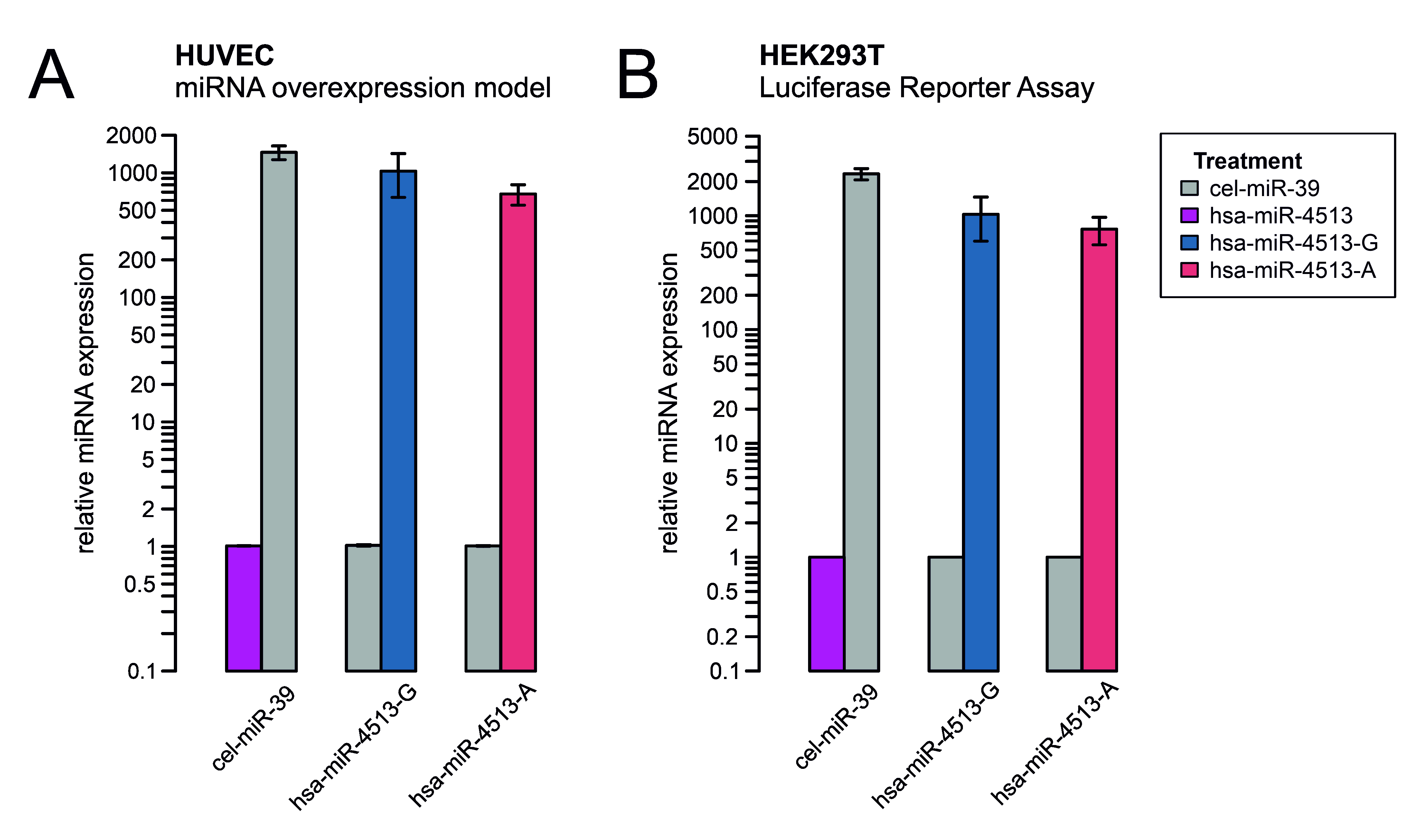


**Figure S2. Relative miRNA expression after transfection.** Transfection efficiency was determined by the relative miRNA expression after transfection in comparison to cells transfected with the respective control miRNA. Cel-miR-39 served as control for hsa-miR-4513-A and hsa-miR-4513-G, while cells transfected with hsa-miR-4513-A and hsa-miR-4513-G served combined as control for cel-miR-39. **(A)** Relative expression 48 h after transfection in HUVECs. Shown are mean values from three independent experiments, with 3 – 6 replicates each. **(B)** Relative expression 24 h after co-transfection with the empty luciferase reporter vector pmirGLO in human embryonic kidney cells (HEK293T). Cells were transfected on 96-well plates and four wells were combined to one sample. Shown are mean values from four independent experiments, with 2 replicates each. Bars indicate standard errors (SE).


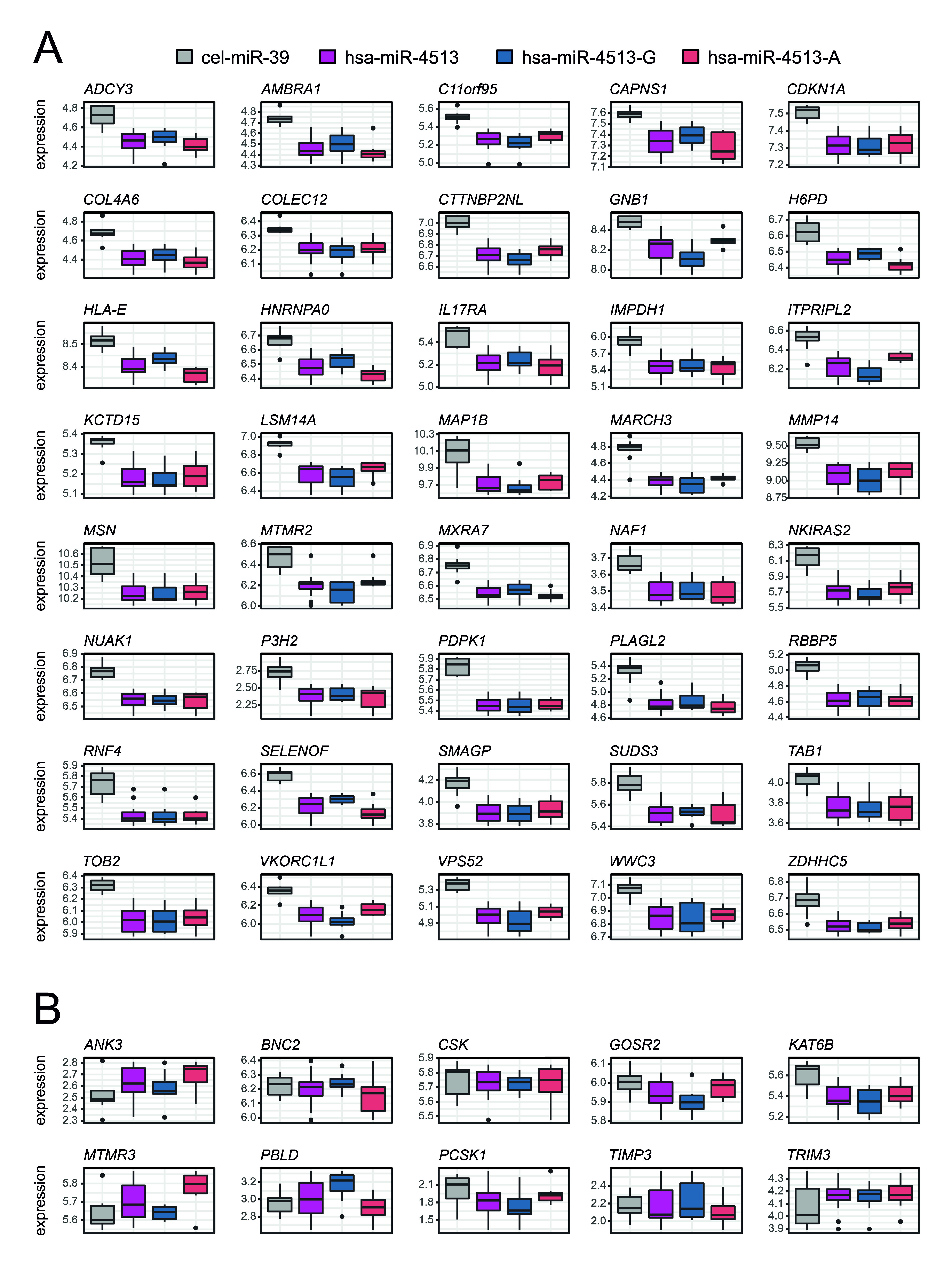


**Figure S3. Target genes of hsa-miR-4513 with combined rs2168518 alleles and previously reported target genes of hsa-miR-4513. (A)** Boxplots represent expression of hsa-miR-4513 target genes identified in the RNA-Seq of HUVECs transfected with hsa-miR-4513-A, hsa-miR-4513-G and cel-miR-39 as control. For a better comparison, samples transfected with hsa-miR-4513-A and hsa-miR-4513-G were combined (shown in purple). In total, 40 genes displayed a significantly decreased expression in hsa-miR-4513 samples compared to control cel-miR-39 samples (false discovery corrected p-value (Q-value) < 0.01). **(B)** Boxplot representations of expression of hsa-miR-4513 target genes previously identified in the literature, as well as the host gene *CSK* in the RNA-Seq data of HUVECs transfected with hsa-miR-4513-A, hsa-miR-4513-G and cel-miR-39 as control. Significant expression differences were observed for *KAT6B* between the control cel-miR-39 and hsa-miR-4513 samples (combined hsa-miR-4513-A and hsa-miR-4513-G samples), as well as for *MTM3* between hsa-miR-4513-A and hsa-miR-4513-G samples (Q-value < 0.05).


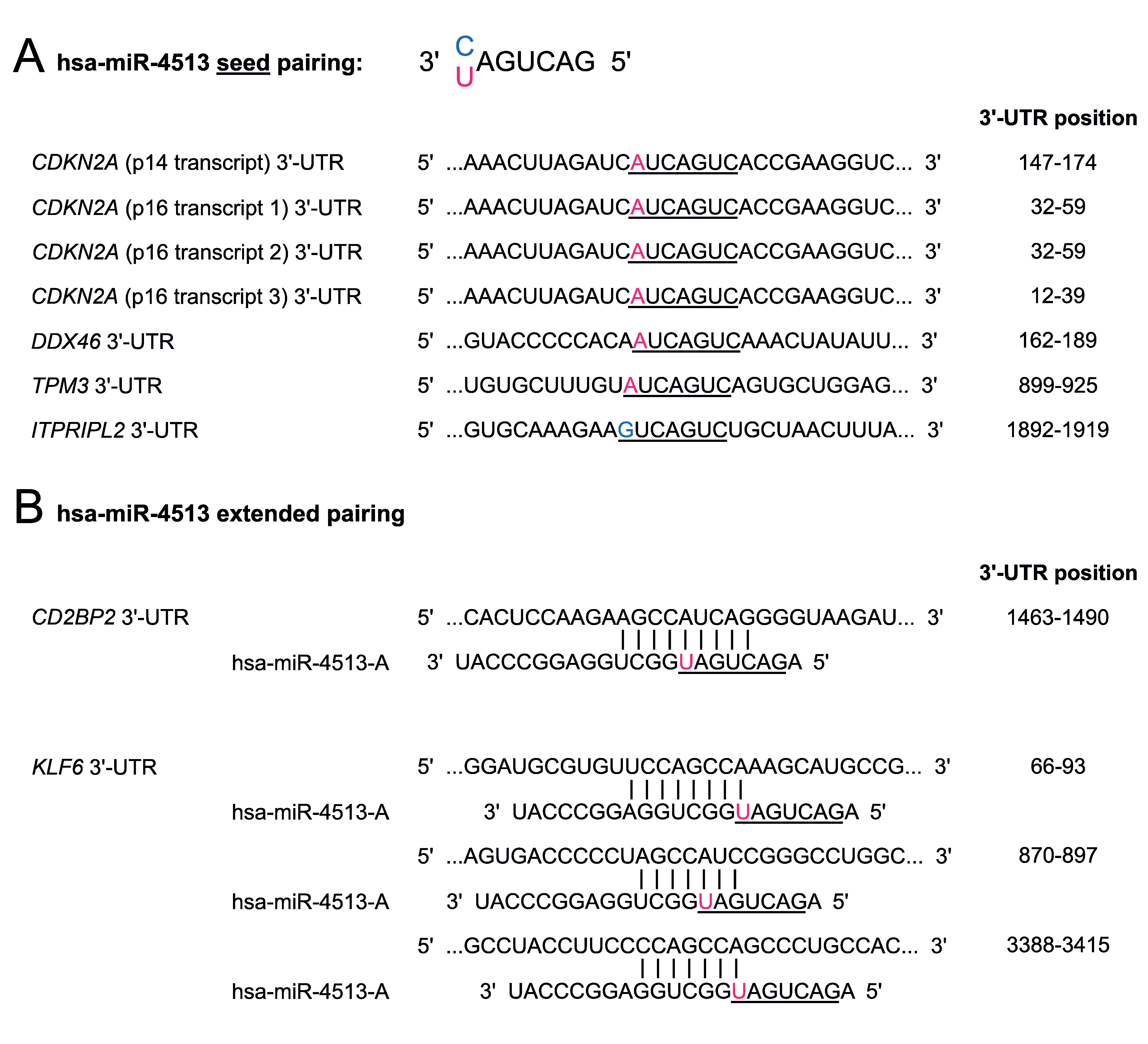


**Figure S4. Allele-specific binding sites of hsa-miR-4513 in the 3’-untranslated regions (3’-UTR) of selected target genes.** Displayed are binding sites of hsa-miR-4513 in the 3’-UTR of target genes which are affected by the seed polymorphism rs2168518. The indicated positions in the 3’-UTR of the transcripts start with 1 for the first nucleotide after the stop codon of the coding sequence. Only binding sites with at least seven nucleotides are shown. **(A)** Binding sites in the 3’-UTR of *CDKN2A*, *DDX46*, *TPM3* and *ITPRIPL2* are perfect seed matches of hsa-miR-4513. Four different transcripts for *CDKN2A* were investigated, one encoding the p14 protein (ENST00000579755) and three encoding the p16 protein (p16 transcript 1: ENST00000304494, p16 transcript 2: ENST00000578845, p16 transcript 3: ENST00000579122). All of those transcripts are present in HUVECs and all contain the same binding site which includes a perfect seed match with hsa-miR-4513-A. **(B)** Not only perfect seed pairing of the miRNA could affect miRNA binding, but also imperfect and extended pairing beside the seed region. In the 3’-UTRs of *CD2BP2* and *KLF6* no perfect seed match with hsa-miR-4513 occurs. Nevertheless, for both genes binding sites for hsa-miR-4513 with at least 7 nucleotides including the position of rs2168518 are present, but shifted towards the 3’ direction of hsa-miR-4513.


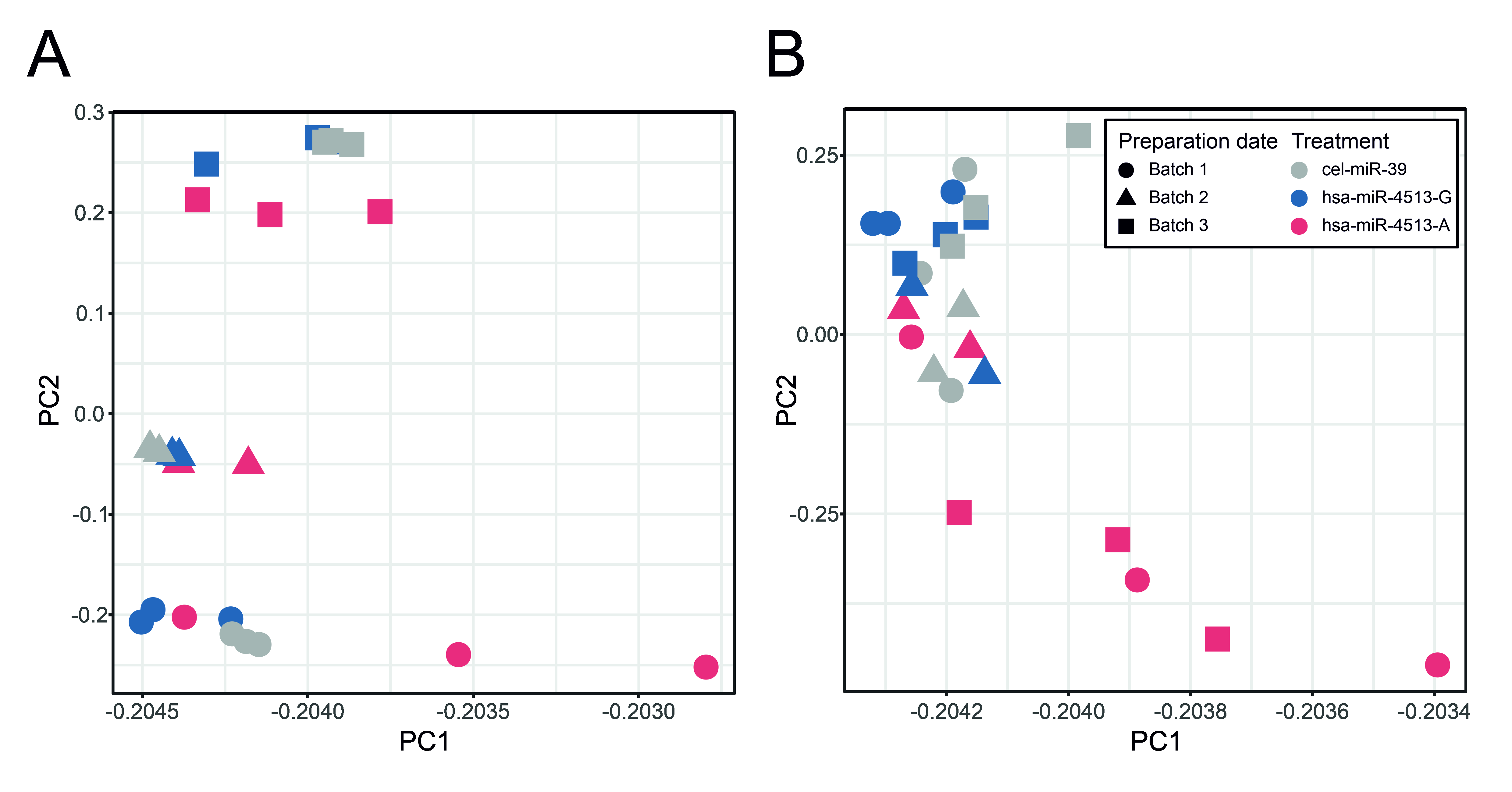


**Figure S5. Principle Component Analysis (PCA) of normalized RNA-Seq data. (A)** A PCA was performed after a first normalization of estimated gene counts with the trimmed mean of M-Values algorithm to identify potential batch effects. Samples clustered according to the RNA isolation date. **(B)** A quantile normalization and ComBat removed those batch effects. PC = principle component.
